# Supplementary figures and images for: Disruption of genes associated with Charcot-Marie-Tooth type 2 lead to common behavioural, cellular and molecular defects in Caenorhabditis elegans
Source: PLoS One. 2020 Apr 15;15(4):e0231600. doi: 10.1371/journal.pone.0231600 (PMC7159224; doi:10.1371/journal.pone.0231600)

Figure S1

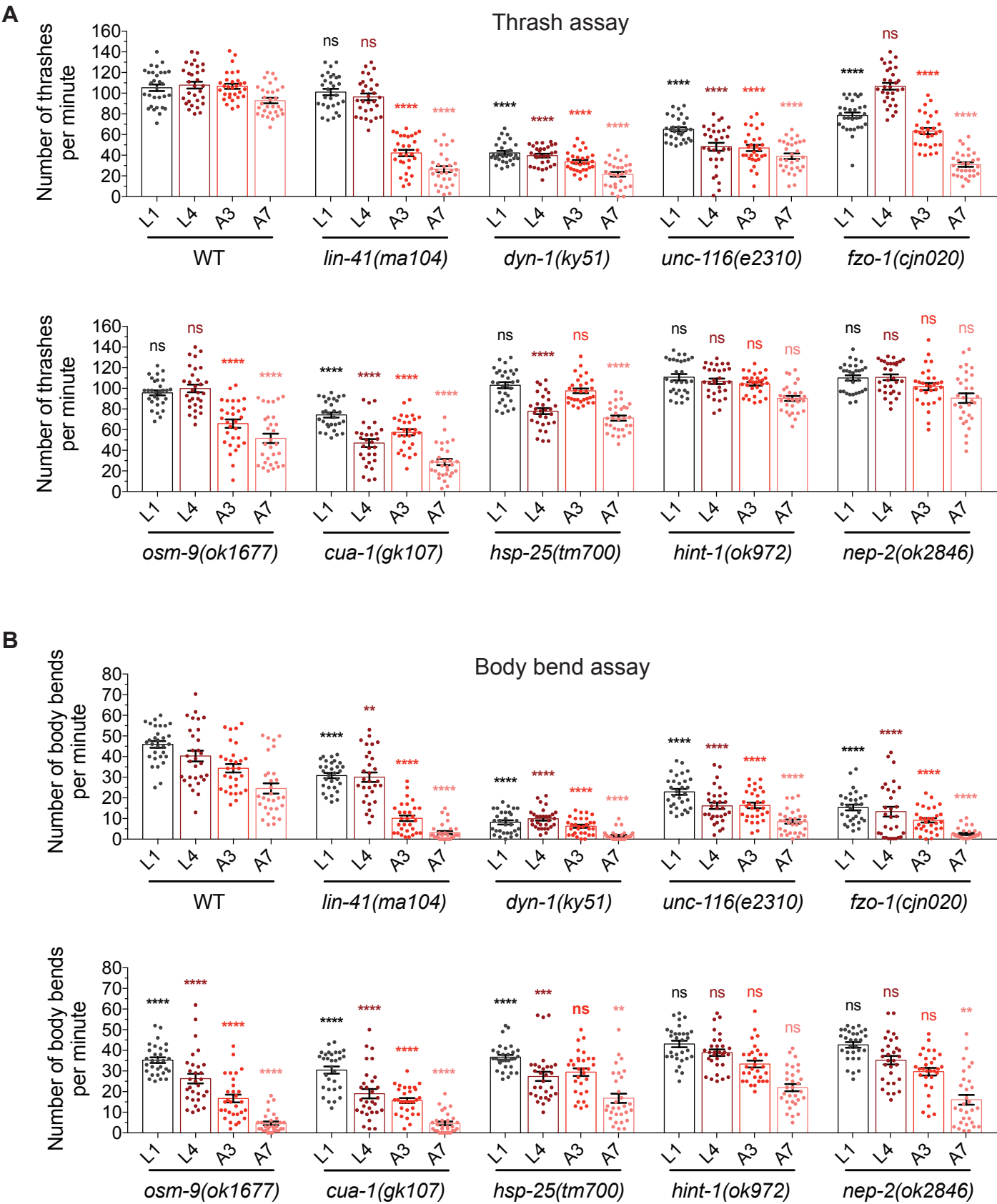

Supplement: S1 Fig — (A) Thrash rates compared between wild-type (WT) and the nine mutant strains, across three different ages, larval stage 1 (L1), larval stage 4 (L4), 3-day old adults (A3) and 7-day old adults (A7). (B) Rate of body bends of WT and CMT2 mutant animals quantified across the same ages as in (A). Each dot in (A) and (B) represents a single animal (n ≥ 30). One-way ANOVA with Dunnett’s post hoc tests were used to compare rates of thrash or body bend between WT and mutant animals in (A) and (B). Data is represented as mean ± S.E.M. **P < 0.01, ***P < 0.001, ****P < 0.0001, ns = not significant. (PDF) [file pone.0231600.s001.pdf]

Figure S2

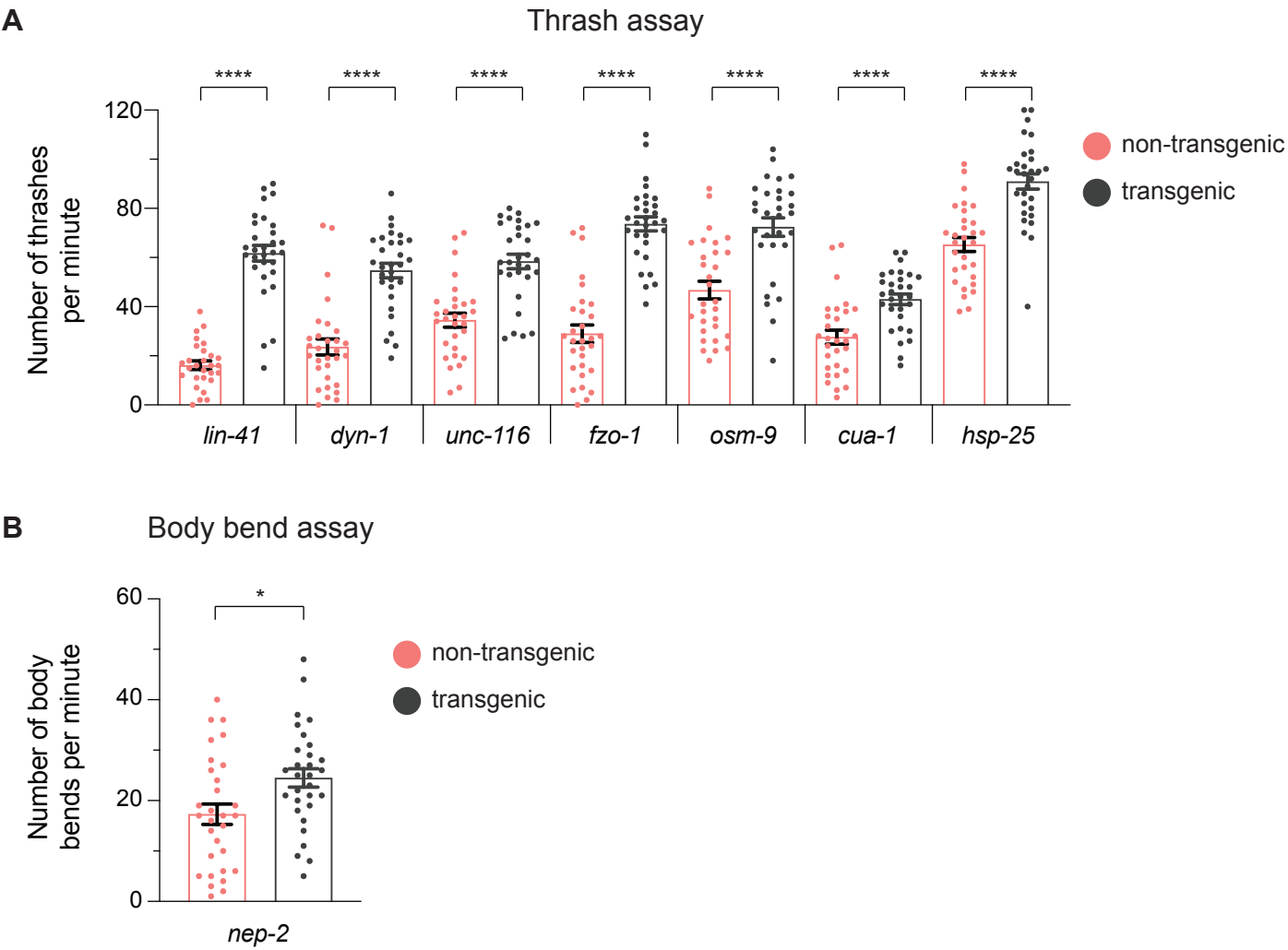

Supplement: S2 Fig — (A) Thrash rates compared between 7-day old adult non-transgenic mutant strains and their transgenic siblings. All transgenic animals carry fosmids containing the corresponding wild-type version of the gene, except for fzo-1, for which a Pmyo-3::fzo-1::unc-54 3`UTR was used, and cua-1, which expresses a Pcua-1::cua-1::cua-1 3’UTR plasmid. Data is represented as mean ± S.E.M, with symbols showing individual animals. (B) Rate of body bends compared between 7-day old adult non-transgenic nep-2 mutants and their transgenic siblings expressing wild-type copies of nep-2 from a fosmid. For (A) and (C), *P < 0.05, ****P < 0.0001 from Student’s t-tests. (PDF) [file pone.0231600.s002.pdf]

Figure S3

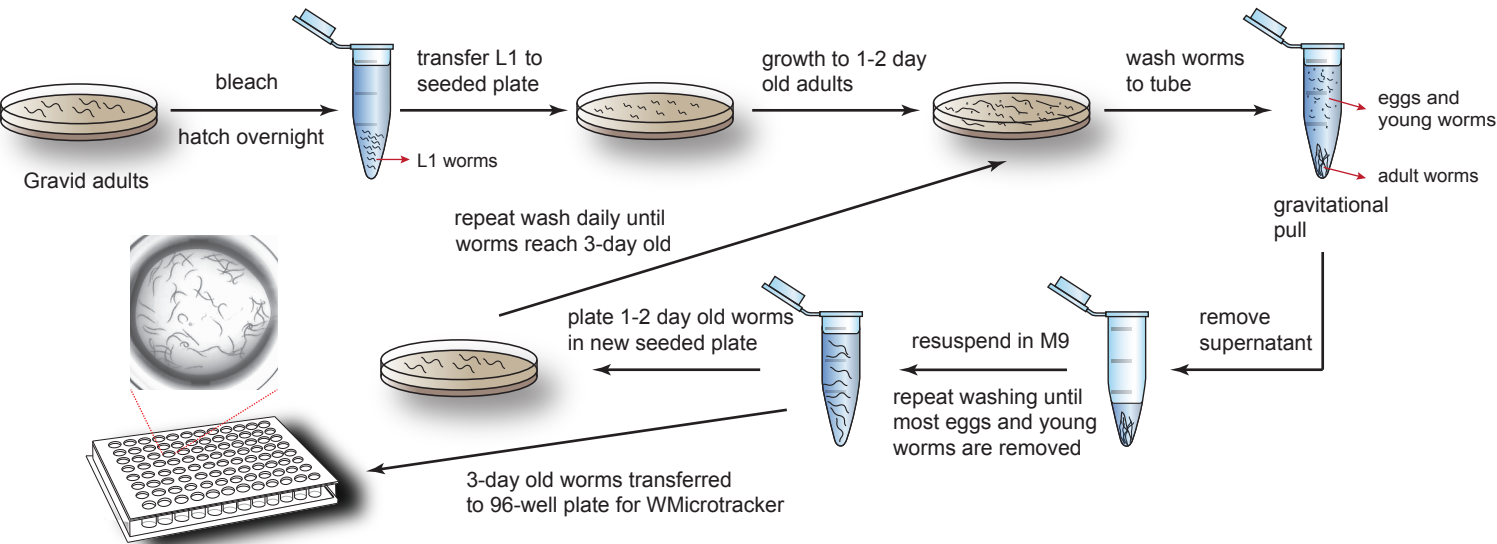

Supplement: S3 Fig — Schematic of the workflow, from synchronization via bleaching and washing, to plating of synchronized 3-day old adult animals in a 96-well plate for experimentation. (PDF) [file pone.0231600.s003.pdf]

**Figure S4**

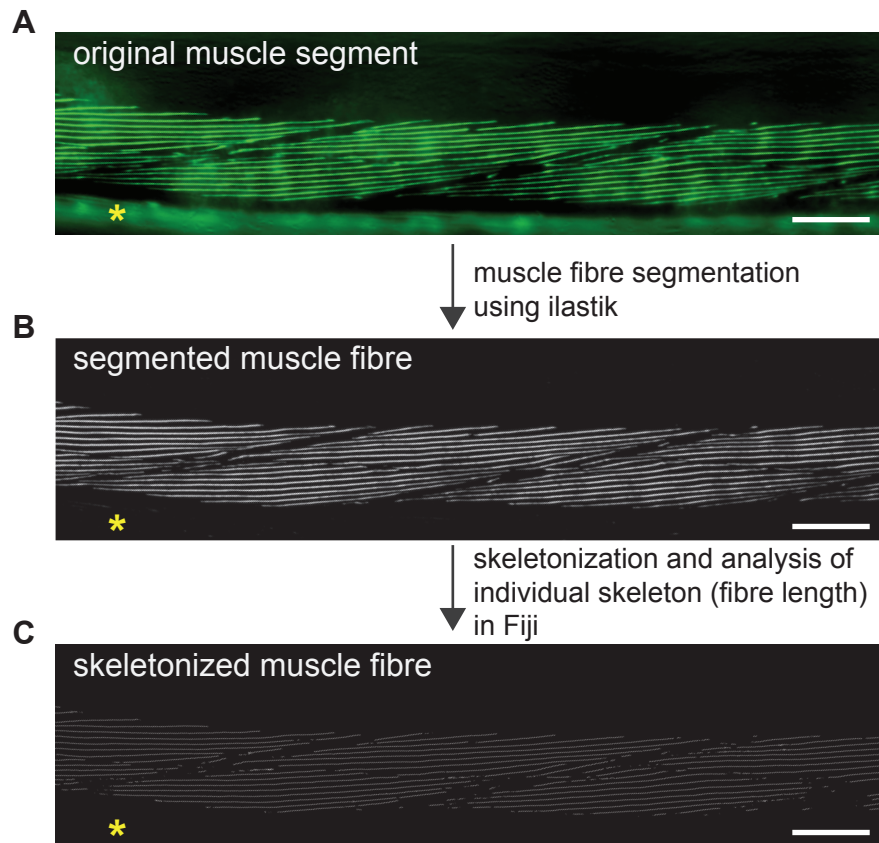

Supplement: S4 Fig — (A) Original muscle segment must contain at least one full visible oblique muscle cell. (B) Distinct myosin fibre is classified and segregated from the unwanted background (in asterisk) in ilastik. (C) Skeletonization of image in Fiji. The image was skeletonized to filter out the border pixels, leaving behind only the skeletal remnants that become the topological representations of the original fibres. Measurement of each fibre was performed following skeletonization. Fibres that were 0 µm or more than 250 µm were excluded. Scale bar represents 25 µm. (PDF) [file pone.0231600.s004.pdf]

**Figure S5**

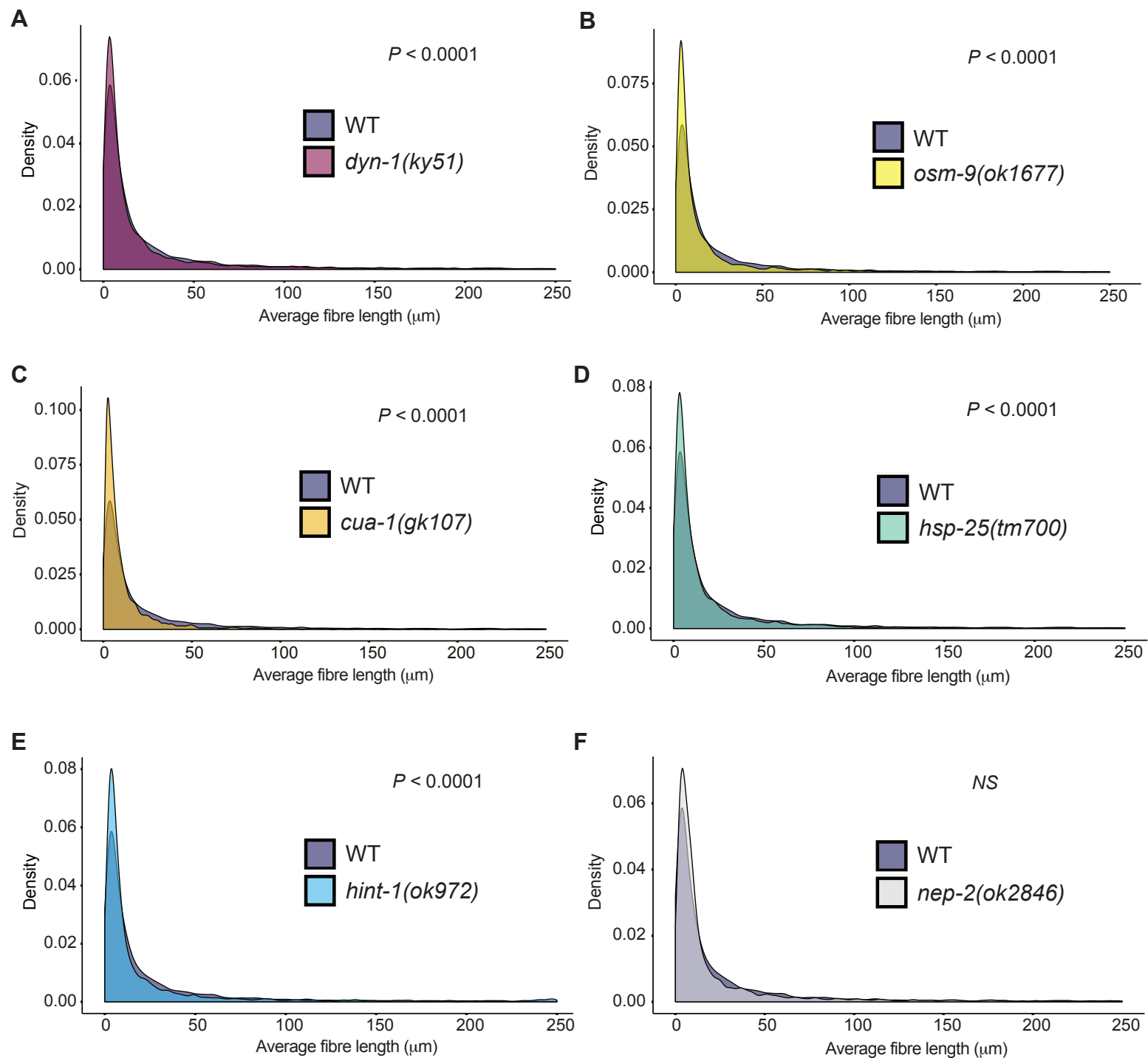

Supplement: S5 Fig — (A-F) The variance of all except for nep-2(ok2846) was statistically different compared to wild-type (WT). Statistics were performed using F test for variances, significance set at P ≤ 0.05. Experiments were performed on 3-day old adult animals that carried stEx30 transgene. (PDF) [file pone.0231600.s005.pdf]
