# Supplementary material for: Disruption of genes associated with Charcot-Marie-Tooth type 2 lead to common behavioural, cellular and molecular defects in Caenorhabditis elegans
Source: PLoS One. 2020 Apr 15;15(4):e0231600. doi: 10.1371/journal.pone.0231600 (PMC7159224; doi:10.1371/journal.pone.0231600)
Supplement: S1 Table — (DOCX) [file pone.0231600.s006.docx]

**Supplementary Table 1**. List of strains used in this study.

| **Strain name** | **Gene (allele)** | **Type of mutation** | **Number of times outcrossed** | **Coded protein (function)** |
| --- | --- | --- | --- | --- |
| RW1596 | *myo-3(st386); stEx30(Pmyo-3::gfp::myo-3 + rol-6(su1006))* | Not annotated  GFP-tagged rescue | - | Myosin heavy chain |
| LX929 | *vsIs48(Punc-17::gfp)* | GFP-tagged WT | - | - |
| BXN418 | *lin-41(ma104)* | Transposon insertion [1] | 3 | RBCC Ubiquitin Ligase (protein degradation, gene regulation) |
| BXN674 | *lin-41(ma104); vsIs48(Punc-17::gfp)* |  |  |  |
| BXN679 | *lin-41(ma104); myo-3(st386); stEx30(Pmyo-3::gfp)* |  |  |  |
| BXN745 | *lin-41(ma104); cjnEx135(lin-41 WRM064dG06 fosmid* | Rescue strain | - | - |
| BXN567 | *dyn-1(ky51)* | Missense substitution (P70S) [2] | 3 | Dynamin GTPase (endocytosis, synaptic vesicle recycling, cytokinesis, degradation of apoptotic cells) |
| BXN678 | *dyn-1(ky51); vsIs48(Punc-17::gfp)* |  |  |  |
| BXN685 | *dyn-1(ky51); myo-3(st386); stEx30(Pmyo-3::gfp)* |  |  |  |
| BXN738 | *dyn-1(ky51); cjnEx139(dyn-1 WRM065aB08*  *fosmid* | Rescue strain | - | - |
| FF41 | *unc-116(e2310)* | Not curated | 15 | Kinesin 1 Heavy Chain (transport and localisation of synaptic vesicle components, axonal transport of neurofilament proteins) |
| BXN675 | *unc-116(e2310); vsIs48(Punc-17::gfp)* |  |  |  |
| BXN680 | *unc-116(e2310); myo-3(st386); stEx30(Pmyo-3::gfp)* |  |  |  |
| BXN737 | *unc-116(e2310); cjnEx141(unc-116 WRM063bE02 fosmid* | Rescue strain | - | - |
| BXN248 | *fzo-1(cjn020); zdIs5* | 2629 bp deletion [3] | 3 | GTPase (outer mitochondrial membrane fusion) |
| BXN623 | *fzo-1(cjn020); vsIs48(Punc-17::gfp)* |  |  |  |
| BXN366 | *fzo-1(cjn020); zdIs5(Pmec-4::GFP); myo-3(st386); stEx30(Pmyo-3::gfp)* |  |  |  |
| BXN336 | *fzo-1(cjn020); zdIs5(Pmec-4::GFP); cjnEx35(Pmyo-3::fzo-1)* | Rescue strain | - | - |
| BXN377 | *osm-9(ok1677)* | 1478 bp deletion [4] | 3 | TRPV channel (osmo- and warmth sensor channel) |
| BXN625 | *osm-9(ok1677); vsIs48(Punc-17::gfp)* |  |  |  |
| BXN628 | *osm-9(ok1677); myo-3(st386); stEx30(Pmyo-3::gfp)* |  |  |  |
| BXN766 | *osm-9(ok1677); cjnEx133(osm-9 WRM065bE12 fosmid* | Rescue strain | - | - |
| BXN562 | *cua-1(gk107)* | 1639 bp deletion [4] | 3 | Copper-transporting P-type ATPase (copper transporter) |
| BXN626 | *cua-1(gk107); vsIs48(Punc-17::gfp)* |  |  |  |
| BXN629 | *cua-1(gk107); myo-3(st386); stEx30(Pmyo-3::gfp)* |  |  |  |
| BXN764 | *cua-1(gk107); cjnEx131(Pcua-1::cua-1::cua-1* 3`UTR | Rescue strain | - | - |
| BXN620 | *hsp-25(tm700)* | 870 bp deletion [4] | 3 | Heat Shock Protein (molecular chaperone, actin organisation, axonal transport of neurofilament proteins) |
| BXN676 | *hsp-25(tm700); vsIs48(Punc-17::gfp)* |  |  |  |
| BXN681 | *hsp-25(tm700); myo-3(st386); stEx30(Pmyo-3::gfp)* |  |  |  |
| BXN740 | *hsp-25(tm700); cjnEx137(hsp-25 WRM063bD07 fosmid* | Rescue strain | - | - |
| BXN619 | *hint-1(ok972)* | 1025 bp deletion [4] | 3 | Histidine Triad Nucleotide Binding Protein 1 (nucleotide hydrolytic activity) |
| BXN624 | *hint-1(ok972); vsIs48(Punc-17::gfp)* |  |  |  |
| BXN627 | *hint-1(ok972); myo-3(st386); stEx30(Pmyo-3::gfp)* |  |  |  |
| BXN542 | *nep-2(ok2846)* | 362 bp deletion [4] | 3 | Neprilysin (signalling peptide regulation) |
| BXN677 | *nep-2(ok2846); vsIs48(Punc-17::gfp)* |  |  |  |
| BXN683 | *nep-2(ok2846); myo-3(st386); stEx30(Pmyo-3::gfp)* |  |  |  |
| BXN736 | *nep-2(ok2846); cjnEx140(nep-2 WRM063bA06 fosmid* | Rescue strain | - | - |

1. Slack FJ, Basson M, Liu Z, Ambros V, Horvitz HR, Ruvkun G. The *lin-41* RBCC gene acts in the *C. elegans* heterochronic pathway between the *let-7* regulatory RNA and the LIN-29 transcription factor. Mol Cell. 2000;5(4):659-69.

2. Clark SG, Shurland DL, Meyerowitz EM, Bargmann CI, van der Bliek AM. A dynamin GTPase mutation causes a rapid and reversible temperature-inducible locomotion defect in *C. elegans*. Proc Natl Acad Sci U S A. 1997;94(19):10438-43.

3. Byrne JJ, Soh MS, Chandhok G, Vijayaraghavan T, Teoh J-S, Crawford S, et al. Disruption of mitochondrial dynamics affects behaviour and lifespan in *Caenorhabditis elegans*. Cellular and Molecular Life Sciences. 2019:1-19. doi: <https://doi.org/10.1007/s00018-019-03024-5>.

4. Deletion Mutant Consortium. Large-scale screening for targeted knockouts in the *Caenorhabditis elegans* genome. G3 (Bethesda). 2012;2(11):1415-25.
